# Enantioselective Behavior of Flumequine Enantiomers and Metabolites' Identification in Sediment

**DOI:** 10.1155/2022/2184024

**Published:** 2022-12-02

**Authors:** Moyong Xue, Xu Gu, Yuchang Qin, Junguo Li, Qingshi Meng, Ming Jia

**Affiliations:** ^1^Institute of Animal Science, Chinese Academy of Agriculture Sciences, Beijing 100193, China; ^2^University of Liege, Functional & Evolutionary Entomology, Agro-Bio-Tech Gembloux 5030, Liege, Belgium; ^3^Feed Research Institute, Chinese Academy of Agricultural Science, Beijing 100081, China

## Abstract

The enantioselective adsorption, degradation, and transformation of flumequine (FLU) enantiomers in sediment were investigated to elucidate the enantioselective environmental behaviors. The results of adsorption test showed that stereoselective differences of FLU enantiomers in sediment samples and the adsorbing capacity of *S*-(−)-FLU and *R*-(+)-FLU are higher than the racemate, and the pH values of the sediment determined the adsorption capacity. Enantioselective degradation behaviors were found under nonsterilized conditions and followed pseudo-first-order kinetic. The *R*-(+)-FLU was preferentially degraded, and there was significant enantioselectivity of the degradation of FLU. It can be concluded that the microorganism was the main reason for the stereoselective degradation in sediments. The physicochemical property of sediments, such as pH value and organic matter content, can affect the degradation rate of FLU. In addition, the process of transformation of FLU enantiomers in water-sediment system had enantioselective behavior, and *R-*(+)-FLU was preferential transformed. Meanwhile, the main metabolites of FLU in the sediment were decarboxylate and dihydroxylation products. This study contributes the evidence of comprehensively assessing the fate and risk of chiral FLU antibiotic and enantioselective behavior in the environment.

## 1. Introduction

In the past several decades, the residues of the veterinary antibiotics used to treat bacterial infections in humans and animals have been extensively detected in various aquatic and sediment environments [1, 2]. Flumequine (FLU), a broad-spectrum antibiotic agent of the fluoroquinolone family, has been commonly used in poultry and aquatic animals especially against Gram-negative bacteria [3]. The main mechanism of FLU is based on inhibiting the nucleic acid synthesis of bacterial action to terminating the normal DNA replication and synthesis [4]. Particularly, FLU is directly applied as a feed additive in aquaculture, which might be retained in the surrounding waters or sediments, owing to their poor bioavailability in aquatic animals. The low bioavailability may result in high concentrations of FLU residues in the aquatic and sediment environments [5].

Approximately 50% of quinolone drugs are chiral compounds and have at least one chiral center in the chemical structure, and most are dispensed and manufactured in the racemic form [6–8]. For many enantiomeric drugs, although the enantiomers have similar physical and chemical properties, they not only produce different pharmacological and toxicological effects but also can be subject enantiospecific metabolism and pharmacokinetic in living systems [9, 10].

**Figure 1:**
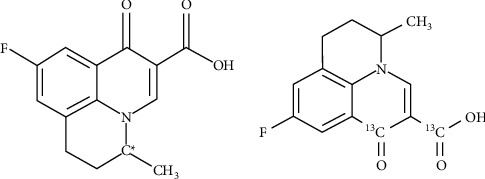
Chemical structure of FLU (C^*∗*^=chiral center) and ^13^C marked position.

FLU has one chiral center and the two enantiomers (Figure 1). Its absolute configuration was confirmed with *S*-(−)-FLU and *R-*(+)-FLU [11]. Studies have shown that the antibacterial activity of FLU enantiomers is significantly different. Wang et al. found that photolysis is the main degradation of FLU in seawater, and the existence of microorganisms led to the difference in degradation of FLU enantiomers [12]. Li studied the stereoselective behaviors of FLU residues in red sea bream after intragastric administration [11]. They found that the half-lives of *S*-(−)-FLU and *R*-(+)-FLU were 12.4 h and 11.2 h, respectively. Plasma concentration of *S*-(−)-FLU is always higher than that of *R*-(+)-FLU.

The degradation behaviors of Rac-FLU in water and sediment are affected by environmental factors such as light [5] and microbial activities [13, 14]. However, the investigations of FLU at the enantiomeric level are limited, especially for some complex matrices such as sediments and the water-sediment system.

In addition, FLU is commonly employed in aquaculture as the racemic form and its enantiomers are frequently ignored, so the risk assessment of FLU in the traditional racemic level is inaccurate [15]. Therefore, it is an important consideration to elucidate the fate, enantioselective behaviors, and ecotoxicological effects of FLU enantiomers in water and the sediment [16, 17].

The aim of this study was, therefore, to identify the environmental behaviors of enrichment, degradation, and transformation and mechanism at the enantiomer level. Simultaneously, its metabolite will be identified in order to understand the fate and effects of FLU enantiomers pollution on the environment, especially in the sediment.

## 2. Materials and Methods

The materials and methods are described in the following sections.

### 2.1. Reagents and Materials

*S*-(−) and *R*-(+)-FLU enantiomers (purity > 90.0%) were obtained from CNW Technologies (Shanghai, China). ^13^C labeled Rac-FLU standard (purity at 99.9%) was obtained from Dr Ehrenstorfer GmbH (Augsburg, Germany). Acetonitrile, methanol, and other organic solvents (HPLC grade) were purchased from Sigma Aldrich (Shanghai, China). All other chemicals and reagents were of analytical grade supplied by Thermo Fisher Scientific (Beijing, China). The HPLC water was prepared through a Milli-Q system (Millipore, MA, USA). The chiral analytical column (Lux 5 *μ*m Cellulose-2, 250 mm × 4.6 mm i.d. × 5 *μ*m) was purchased from Phenomenex (American). The 0.22 *μ*m Filter Unit and Cleanert PEP Solid phase extraction cartridges (500 mg/6 mL) were purchased from Bonna-Agela Technologies (Beijing, China).

### 2.2. Instruments

A 5600 accurate mass tandem quadrupole-time-of-flight (Q-TOF) mass spectrometer (AB SCIEX) was used to quantify FLU enantiomers. Other equipment used were an electronic balance (Mettler-Toledo, Switzerland), automatic solid phase extractor (Reeko Instrument Co., Ltd, China), and nitrogen-blowing concentrator (TongTaiLian Technology Co., Ltd, China).

### 2.3. Preparation of Standard Stock Solutions

Standard solutions of Rac-FLU and *S*-(−) and *R*-(+)-FLU enantiomers were prepared in pure ACN to achieve a final concentration of 200.0 mg/L. All solutions were protected against light and stored in the dark at 4°C.

### 2.4. Sediment Sample Collection and Preparation

Sediment samples were obtained from three different pools (0–20 cm surface layer) in Jinghai District, Tianjin City, China, using the bottom sampler to collect sediment samples at the bottom of pools. All sediment samples were randomly collected in triplicate from an area of approximately 1 m^2^ in the center of each sediment site. All samples were refrigerated in storage at 4°C and returned to the laboratory. The sediment samples were air dried first, and the particle size refers to the original particle size. The physicochemical properties of sampled sediments are listed in Table 1. These samples did not contain the target analytes. After the natural drying process, the sediment samples were homogenized into powder and were passed through the mesh sieve and stored in the refrigerator at −20°C until analysis. The sample collection and preparation progress are similar to our previously published report [18].

### 2.5. Adsorption Experiment

The background solution was prepared with 0.005 mol/L CaCl_2_ (maintaining ion concentration) and 100 mg/L NaN_3_ (inhibiting microbial activity). Rac-FLU and *S*-(−) and *R*-(+)-FLU enantiomers were added to the background solution, respectively. Finally, the concentration of FLU solution used in the experiment was 20 mg/L.

A total of 20 mL FLU solution and 1.0 g sediment sample (dry weight) were added to 50 mL centrifuge tubes with a screw cap. All the centrifuge tubes were shaken at 25°C in a temperature-controlled shaking incubator at a shaking speed of 230 rpm. The shaking incubator was covered with a black cloth, and all procedures were conducted in the dark to avoid photodegradation. Sampling after shaking starts at 30 min, 1 h, 2 h, 8 h, 12 h, 24 h, 2 d, 3 d, 4 d, 5 d, 6 d, 7 d, 8 d, 9 d, and 10 d. Then, these samples were centrifuged at 8000 rpm for 10 min. The supernatant was then filtered through a 0.22 *μ*m syringe filter before Q-TOF/MS analysis. All experiments were conducted in triplicates. Blank samples contain the same concentration of FLU and a total background solution volume of 20 mL (without sediment). The procedure was consistent with the above.

The amount of FLU adsorbed to the sediment was calculated as

(1) $$\begin{array}{c} Cs=\left(Ci-Ce\right)\times \frac{V}{M}\text{,} \end{array}$$

where *Cs* (mg/kg) is the uptake amount of the FLU at equilibrium, *Ci* (mg/L) and *Ce* (mg/L) are the initial and equilibrium concentrations of FLU in solution, *M*(*g*) is the mass of the sorbent, and *V*(*L*) is the volume of the solution.

### 2.6. Degradation Experiment

The degradation experiment of FLU enantiomers were examined under both sterilized and nonsterilized condition in three different sediments. The sterilized experiment represents abiotic degradation only, 250 g sediment (dry weight) was weighed into 500 mL conical flask bottles, and the sediment was sterilized at 120°C for 15 min and then poured into 125 mL sterile water prior to the addition of the FLU enantiomers. The sediment-to-solution ratios adopted were 2 : 1 (2 g of sediment to 1 mL of solution). The initial concentration of Rac-FLU and *S*-(−) and *R*-(+)-FLU enantiomers (20 mg/kg) was used by adding into each conical flask, respectively. Both sterilized and nonsterilized conical flasks were sealed with cotton wool. After that, they were put into a thermotank at 35°C and prevented from light exposure (the sterilized group were placed in a sterile thermotank). For both sterilized and nonsterilized degradation experiments, samples were collected on 0, 3, 7, 14, 28, 42, 56, 70, 84, 98, 128, 158, and 188 d after treatment and stored at −80°C until analysis. All experiments were conducted in triplicates.

For the nonsterilized experiment, the sediment and water used in experiment were not sterilized. Other experimental procedures are the same as the sterilized experiment.

Data from the degradation experiments were fitted to the first-order equations:

(2) $$\begin{array}{c} Ct=C_{0}e^{-kt}\text{,} \end{array}$$

where *C*_*t*_ is the concentrations of antibiotics (mg/kg) for time *t* (days), *C*_0_ is the initial antibiotics concentration (mg/kg), and *k* is the degradation coefficient. Half-lives (*t*_1/2_, *d*) were calculated by the equation: *t*_1/2_=ln 2/*k*.

### 2.7. Transformation Experiment

Sediments can serve as a source in processes involving the migration and transformation of antibiotics [19]. Two rectangle water tanks were used to perform the migration and transformation experiment, and the tank is made of glass in order to reduce the sorption of antibiotics. The tanks were housed in a large laboratory, with the temperature of the room kept at 20 ± 2°C. About 2 kg of sediment were laid evenly at the bottom of the tank, and 3 kg of water were then slowly added to the tank, about 50 mm above the surface of sediment. Then, 20 mg/kg of Rac-FLU antibiotics were dissolved and spiked into the water. 5 g of sediment samples were accurately weighed and collected in different sampling periods to observe the migration of the FLU antibiotics from water to sediment, and these results could be useful for assessing the migration and fate of commonly used antibiotics in water-sediment systems.

### 2.8. Sample Extraction and Purification

The FLU enantiomers in sediment and water were determined according to the procedures described in our previous study [18]. Briefly, dry sediment samples (2.00 ± 0.01 g) were weighed into a 50 mL centrifuge tube, and then these sediment samples were extracted three times with 30 mL ACN and EDTA-Mcllvaine buffer solution (40 : 60, v/v). The extract solution for each sample was evaporated and diluted to 30 mL with Milli-Q water.

The extracts were then passed through Cleanert PEP (polar enhanced polymer) cartridges for purification. The analytes were eluted from each cartridge with 6 mL methanol and dried under a gentle nitrogen stream. Then the resultant residue was finally redissolved in 1 mL methanol and filtered through a 0.22 *μ*m filter for HPLC-Q-TOF/MS analysis and quantification.

### 2.9. Enantiomer Q-TOF-MS Determination

The chromatographic analysis of the FLU enantiomers was performed on an accurate mass tandem quadrupole-time-of-flight (Q-TOF) mass spectrometer with a chiral Lux Cellulose-2 column. The mobile phase consisted of 0.2% acetic acid in water as solvent A and acetonitrile as solvent B. The gradient elution program was as follows: 0–20 min, A : B (45 : 55, V/V); 20–24 min, A : B (5 : 95, V/V); and 24–25 min, A : B (45 : 55, V/V). The injected volume was set at 1 *μ*L, and the total run time was 30 min at a flow of 1 mL/min [18].

The enantiomeric fraction (EF) was used to measure the enantioselectivity of FLU in the sediment during these experiments. The EF was described by the following equation:

(3) $$\begin{array}{c} EF=peak\;areas\;of\;\frac{R}{\left(R+S\right)}\text{.} \end{array}$$

The EF value ranges from 0 to 1, and EF = 0.5 represents the racemate.

## 3. Results and Discussion

The results and discussion of the study are explained in the following sections.

### 3.1. Method Validation

The rates of recovery values ranged from 71.7 ± 12.5% to 84.6 ± 5.6% for both FLU enantiomers in the sediment. The LOQs were 8.0 *μ*g/L for two enantiomers. Eight concentrations (1, 2, 5, 10, 20, 50, 100, and 200 *μ*g/L) of each FLU enantiomers were used to construct the calibration curves (*R*^2^ > 0.99). The details of analytical method validation are summarized in the supplementary file (available here) and also described in our previous study [18].

### 3.2. Enantioselective Adsorption of FLU in the Sediment

**Figure 2:**
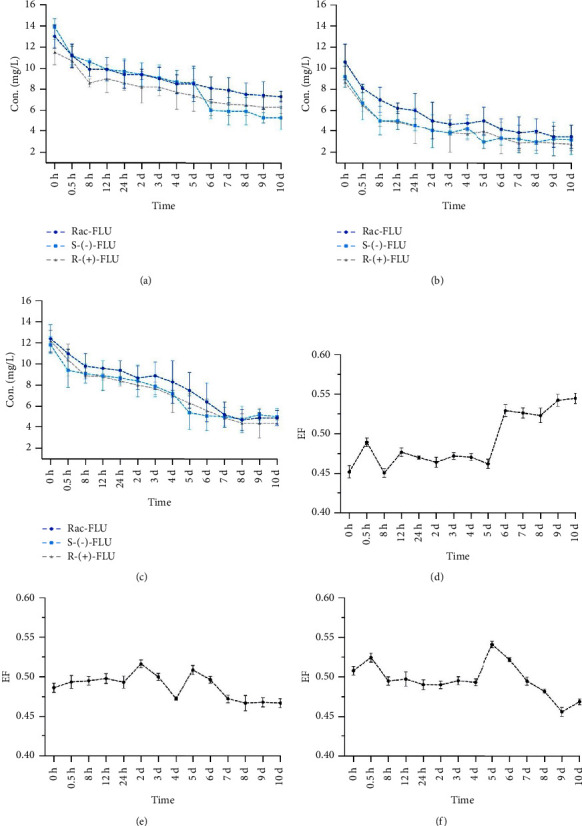
Adsorption curves in 1# (a), 2# (b), and 3# (c) and EF variation in 1# (d), 2# (e), and 3# (f) of FLU in three different sediments. The points and error bars represent the mean and standard deviation of replicates, respectively (*n* = 3).

The changes over time in the concentration of Rac-FLU and FLU enantiomers in three different sediments are shown in Figures 2(a)–2(c). The original spiked concentrations of Rac-FLU and each FLU enantiomers were 20 mg/L. However, the concentrations in sediment of all antibiotics, detected at the first sampling event, were much lower than the initial spiked concentrations because of the rapid adsorption to suspended particles and sediment.

These results indicated that enantioselectivity existed during the adsorption of FLU enantiomers in 1# sediment (Figure 2(d)). In the early stage of the adsorption period, EF values were all below 0.5, so the *R*-(+)-FLU adsorbed faster than the *S*-(−)-FLU during this period. After 5 d of the adsorption period, the *S*-(−)-FLU adsorbed faster than the *R*-(+)-FLU.

Besides, the EF values fluctuated around 0.5 during the whole adsorption period (Figure 2(e)). Therefore, the adsorption behaviors of FLU enantiomers had no enantioselectivity in 2# sediment.

These results of Table 2 and Figure 2(f) indicate that enantioselectivity existed during the adsorption of FLU enantiomers in 3# sediment. There was significant difference in the adsorption capacity of Rac-FLU and *R*-(+)-FLU (*P* < 0.05). In the early stage of the adsorption period, there was no obvious enantioselectivity of FLU enantiomers. After 5 d of the adsorption period, the *R*-(+)-FLU adsorbed faster than the *S*-(−)-FLU. These results indicated that the enrichment of one FLU enantiomer entering the environment [20].

Many studies have shown that the adsorption capacity of antibiotics in the sediment may be affected by the pH value of different sediments [21, 22]. The higher the pH value, the lower the adsorption capacity of antibiotics in sediments. This is mainly because the adsorption of antibiotics is related to the charged state of sediments, and pH value can substantially contribute to the adsorption behavior by changing the charge state of antibiotics [23–25]. In the view of the obtained results, Table 1 shows that 2# sediment had the lowest pH value; however, the adsorption capacity of FLU in 2# sediment was significantly stronger than the 1# and 3# sediments. Besides, enantioselectivity existed during the adsorption of FLU enantiomers in 1# and 3# sediments, so the stereoselective adsorption differences of FLU enantiomers in sediments is also related to the pH value of sediments.

### 3.3. Enantioselective Degradation of FLU in the Sediment under Sterile Condition

**Figure 3:**
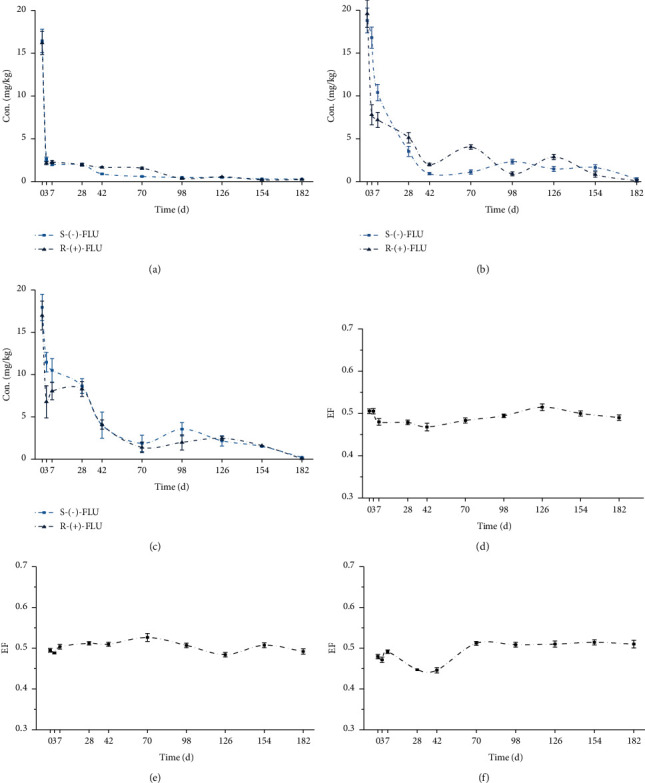
Degradation curves in 1# (a), 2# (b), and 3# (c) and EF variation in 1# (d), 2# (e), and 3# (f) of FLU in the three different sediments under sterile conditions. The points and error bars represent the mean and standard deviation of replicates, respectively (*n* = 3).

The degradation of the FLU enantiomers in three different sediments showed first-order kinetic behavior, with the correlation coefficient values (*R*^2^) between 0.7235 and 0.9135 (Table 3). The degradation curves of FLU enantiomers were given in Figures 3(a)–3(c), and the data show that both *R*-(+)-FLU and *S*-(−)-FLU degraded over time and both enantiomers disappeared at similar rates in three different sediments under sterile conditions.

In the whole adsorption period of 2# sediment, the adsorption capacity of *S*-(−)-FLU and *R*-(+)-FLU are higher than the Rac-FLU (Table 2). There were significant differences in the adsorption capacity of *S*-(−)-FLU, *R*-(+)-FLU and Rac-FLU (*P* < 0.05), but there were no significant differences between *S*-(−)-FLU and *R*-(+)-FLU (*P* > 0.05).

As shown in Table 3, the degradation of FLU enantiomers in 2# sediment (*t*_1/2_ = 39.38 days for *S*-(−)-FLU, 34.31 days for *R*-(+)-FLU) was slightly faster than those of other sediments. Table 1 shows the lowest pH (7.03) and lowest organic content (10.9687 g/kg) in 2# sediment; therefore, it can be speculated that the pH value and organic content in the sediment were the factors affecting the degradation rate of FLU enantiomers in sterile condition. More importantly, the *R*-(+)-FLU degraded more rapidly than *S*-(−)-FLU in three sediments.

In the three kinds of test sediments, the EF values (Figures 3(d)–3(f)) were nearly 0.5 during the whole period. It can make a conclusion that *R*-(+)-FLU and *S*-(−)-FLU degradation were not enantioselective in the sediment under sterilized condition due to no microbial activity. Thus, microbial decomposition can play an important role in stereoselective metabolism of FLU degradation in the three sediments.

### 3.4. Enantioselective Degradation of FLU in the Sediment under Natural Condition

**Figure 4:**
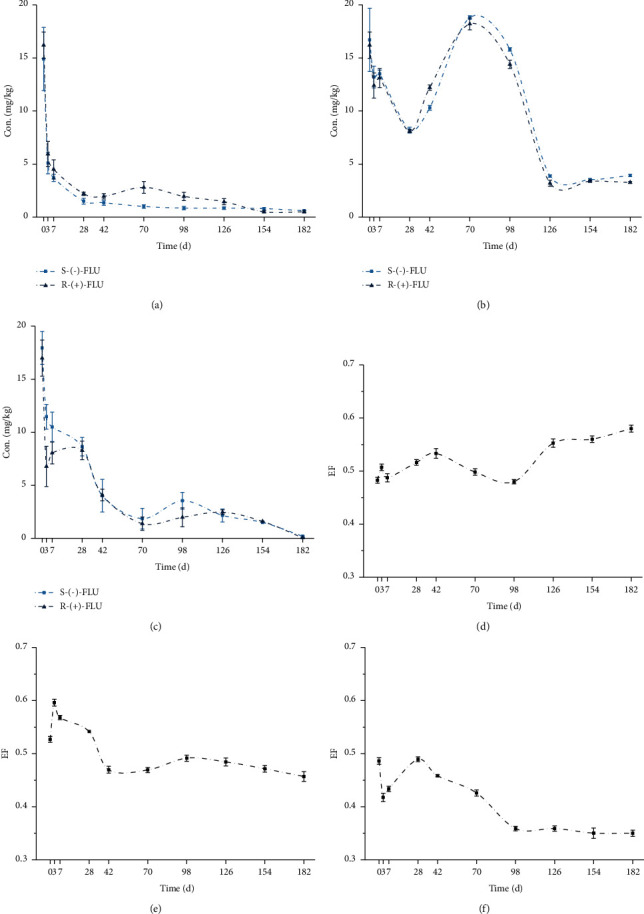
Degradation curves in 1# (a), 2# (b), and 3# (c) and EF variation in 1# (d), 2# (e), and 3# (f) of FLU in the three different sediments under natural conditions. The points and error bars represent the mean and standard deviation of replicates, respectively (*n* = 3).

Figures 4(a)–4(c) show the degradation curves of both FLU enantiomers under natural conditions in the three different sediments, and it can be seen that both enantiomers disappeared over time. However, in 2# sediment, FLU enantiomers were degraded to about 10 mg/L, and then, the concentration of enantiomers increased significantly after 56 days of degradation. After that, the concentration of both enantiomers dropped to 3 mg/L. As it is well known, the environmental sediments are very complex and they have different compositions and present high variability [26]. So, the microorganism action and differences in the composition of sediments could play a role in this change [22]. Therefore, except for 2# sediment, the degradation of both FLU enantiomers in 1# and 3# sediment under natural conditions followed first-order kinetics with *R*^2^ ranging from 0.8017 to 0.8875 (Table 3), and the first-order rate constants were derived from ln(*C*_0_/*C*) versus *t* plots by regression analysis for each experiment.

The enantiomers have the similar half-life in 1# and 3# sediments; however, the observed differences of the half-life in 2# sediment (*t*_1/2_ = 91.18 days for *S*-(−)-FLU, 82.50 days for *R*-(+)-FLU) may be determined by the complex organic matrix and pH value. Compared with the half-life of FLU enantiomers in sterile condition, a slower dissipation of FLU enantiomers in sediments under natural condition was observed.

The EF values (Figures 4(d)–4(f)) showed that enantioselectivity existed during the degradation process of FLU enantiomers in different sediments. There was an increasing trend of EF value with time in 1# sediment that indicate the *S*-(−)-FLU degraded more rapidly. However, the EFs were under 0.5 (after 28 days in 2# sediment) in 2# and 3# sediment and decreased with time. The data suggest the slow degradation of *S*-(−)-FLU. The enantioselective degradation rate of FLU enantiomers is different between three different sediments probably because the chemical or physical activities of high organic matter in 1# sediment.

It is clear that microbial activities played a major role in enantioselective degradation of FLU. Moreover, the organic content of sediments is important to explain the differences in the degradation behavior, and the pH value probably plays an important role in enantioselectivity of FLU enantiomers across different sediments [21, 22, 27].

In addition, the structure of chiral compounds is not stable, so more research had been done to clarify whether there are underlying processes of enantiomeric inversion and transformation in the environment. The *S-*(−)-FLU (or *R*-(+)-FLU) was, respectively, added into the sediment, and the results showed that no *R-*(+)-FLU (or *S*-(−)-FLU) was detected at any time during the whole degradation process under natural or sterilized conditions.

### 3.5. Enantioselective Transformation of FLU in the Water-Sediment System

**Figure 5:**
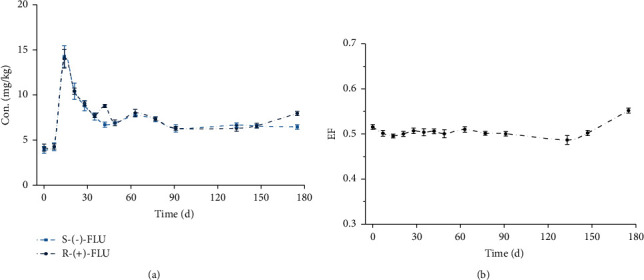
Temporal changes of FLU enantiomers in the bottom sediment of the tank system. The points and error bars represent the mean and standard deviation of replicates, respectively (*n* = 4).

The change over time in the concentration of FLU in the sediment of the water-sediment system is shown in Figure 5(a). The original spiked concentrations of the FLU in the overlying water were 20 mg/kg. The concentration of the sediment of FLU, detected at the earlier sampling event (7 days), was much lower than the initial spiked concentrations. However, because of the rapid sorption to suspended particles and sediment, the concentration of FLU in the sediment rapidly increased. Concentration profiles in the overlying water and sediment suggested that the diffusive transfer of FLU into the sediment was a quick process, with the FLU enantiomers generally detected in the sediment at a maximum concentration about 14 mg/kg at a very short sampling interval. After that, the degradation was observed during the experiment period, and this may be attributed to microbial degradation. These results also suggest that the sediment can potentially act as a significant secondary source of antibiotics that can be released into water [28, 29].

The EF values (around 0.5) in Figure 5(b) show that the transformation behavior of FLU enantiomers had no enantioselectivity in the water-sediment system before 150 days. However, the stereoselective transformation behavior occurred after 150 days because of an increase in the EF values' level. The results indicated that the transformation of FLU enantiomers in the water-sediment system had enantioselective behavior, and *R-*(+)-FLU transformed faster than *S*-(−)-FLU.

### 3.6. Main Metabolites of FLU Identification

**Figure 6:**
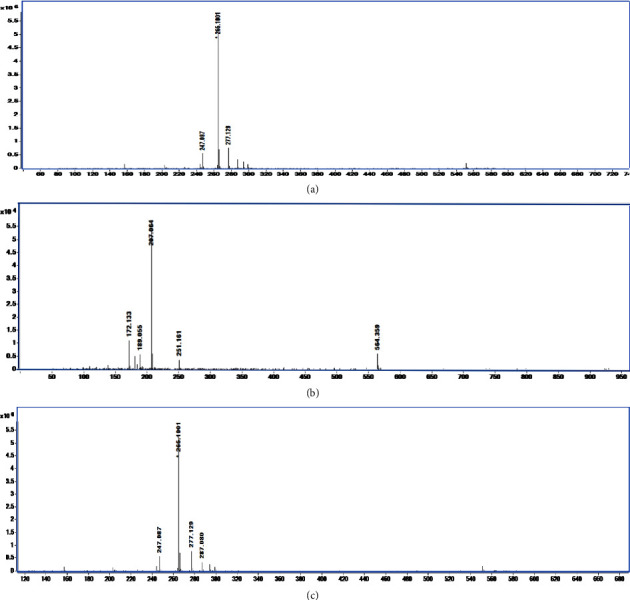
MS/MS spectra of ^13^C FLU (a), MS/MS spectra of metabolites *m*/*z* 207 (b), and MS/MS spectra of metabolites *m*/*z* 247 (c) (abscissa: counts vs. mass-to-charge *m*/*z*; ordinate: intensity).

Identification of molecular ions representing possible metabolites is an indispensable step in the overall identification procedure of drug metabolites using LC/MS/MS approaches [30]. ^13^C labeled FLU in sediment samples were analyzed. We obtained fragmentation patterns, showing intense ion at *m*/*z* 265 (^13^C-FLU), *m*/*z* 207, and *m*/*z* 247 (Figures 6(a)–6(c)).

**Figure 7:**
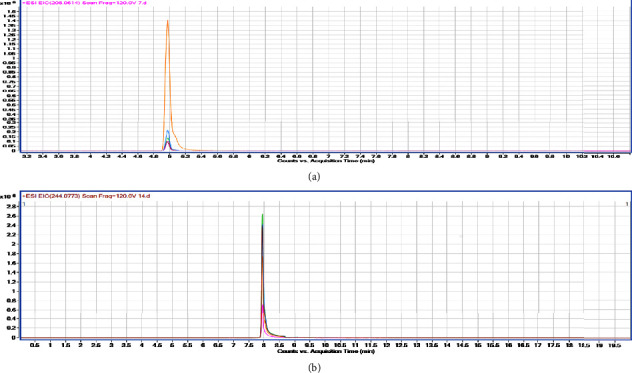
Chromatogram of metabolites *m*/*z* 207. (a) Chromatogram of metabolites *m*/*z* 247 (b).

Figure 7(a) describes the concentration of *m*/*z* 207 (265-COOH) metabolite increased during the experiment period. The content of *m*/*z* 247 (265-OH) metabolite rapidly increased and then gradually declined.  Figure 7(b) shows that the metabolite degradation maybe due to the microorganism action. These were demonstrated that the main metabolites of FLU in the sediment were decarboxylate and dehydroxylation.

## 4. Conclusion

In the present study, a chiral residue analysis method was successfully used to the study of enantioselective adsorption, degradation, and transformation behaviors of FLU enantiomers in different sediments. The results indicated that the FLU enantiomers generated stereoselective behavior in the adsorption of sediment, and the adsorption capacity of *R-*(+)-FLU and *S*-(−)-FLU were much higher than the Rac-FLU in three different sediments; meanwhile, there was significant difference in the adsorption capacity between Rac-FLU and *R-*(+)-FLU or *S*-(−)-FLU. The pH value of the sediment had an influence on the adsorption capacity and enantioselective adsorption of FLU.

Through the degradation test, the degradation of FLU in the sterilized sediment would not be enantioselective. The degradation of FLU enantiomers complied with first-order kinetics and showed stereoselective under nonsterilized condition, which demonstrated that the *R*-(+)-FLU degraded faster than *S*-(−)-FLU. Besides, the degradation rates of both FLU enantiomers were different under sterile and natural conditions. These results indicated that stereoselective degradation and enantioselective differences of FLU enantiomers may depend on the pH and organic content when different microorganisms are involved in the sediment [31]. In addition, stereoselective behavior also occurred in the transformation of FLU in the water-sediment system, and *R-*(+)-FLU transformed faster from water to sediment. Furthermore, the main metabolites of FLU in the sediment were decarboxylate and dehydroxylation products.

These results might be helpful to evaluate the environmental behaviors of chiral FLU, providing the basic data for the evaluation of environmental and ecological risk assessment and the rational suggestions for optically pure antibiotic development and application.

## Figures and Tables

**Table 1:** Properties and locations of different sediments.

Sample number	pH value	Organic content (g/kg)	Grain size (%)			Sampling site
			0.01–2.00 (*μ*m)	2.00–50.00 (*μ*m)	50.00–2000.0 (*μ*m)	
1#	8.32	49.6358	41.105	58.895	0	Tianjin, Jing'an district
2#	7.03	10.9687	18.464	77.034	4.502	Tianjin, Wuqing district
3#	8.75	15.4507	19.019	77.648	3.333	Tianjin, Hexi district

**Table 2:** Adsorption capacity (mg/kg) of FLU enantiomers in different sediment samples. Error bars are the standard deviations of the means of adsorption tests on three replicates.

	1#	2#	3#
Rac-FLU	135.0 ± 1.2^a^	164.0 ± 0.8^a^	149.0 ± 14.6^a^
*S*-(−)-FLU	165.6 ± 6.1^c^	173.2 ± 0.4^b^	161.4 ± 1.3^ab^
*R*-(+)-FLU	154.6 ± 1.5^b^	172.6 ± 2.7^b^	169.6 ± 0.1^b^

^a,b,c,^ within the same column, the same superscripts denoted no significant difference (*P* > 0.05), and different superscripts denoted significant difference (*P* < 0.05). ^1,2,3^ represent the three different sediment samples.

**Table 3:** The degradation of kinetic equations and half-life period under sterile and natural conditions.

Sediment	FLU	Sterile conditions			Natural conditions		
		Kinetic equations	*R* ^2^	*t*_1/2_(*d*)	Kinetic equations	*R* ^2^	*t*_1/2_(*d*)
1*#*	*S*-(−)-FLU	*Y* = −0.0160*x* − 1.7932	0.7235	43.31	*Y* = −0.0127*x* − 1.5467	0.8168	54.57
	*R*-(+)-FLU	*Y* = −0.0177*x* − 1.6003	0.7863	39.15	*Y* = −0.0144*x* − 1.1550	0.8017	48.13

2*#*	*S*-(−)-FLU	*Y* = −0.0176*x* − 0.5709	0.8059	39.38	*Y* = −0.0076*x* − 0.2486	0.5830	91.18
	*R*-(+)-FLU	*Y* = −0.0202*x* − 0.6548	0.7757	34.31	*Y* = −0.0084*x* − 0.2404	0.6223	82.50

3*#*	*S*-(−)-FLU	*Y* = −0.0174*x* − 0.4680	0.9063	39.83	*Y* = −0.0121*x* − 0.7678	0.8045	57.27
	*R*-(+)-FLU	*Y* = −0.0182*x* − 0.3692	0.9135	38.08	*Y* = −0.0141*x* − 0.5311	0.8875	49.15

*R* ^2^, determination coefficient.

## Data Availability

The data used to support the findings of the study can be obtained from the corresponding author upon request.

## Supplementary Materials

Supplementary Materials The description of the supplementary materials is as follows: This is the detailed information of method validation and method optimization.
