# Supplementary material for: Enantioselective Behavior of Flumequine Enantiomers and Metabolites' Identification in Sediment
Source: J Anal Methods Chem. 2022 Dec 2;2022:2184024. doi: 10.1155/2022/2184024 (PMC9733987; doi:10.1155/2022/2184024)
Supplement: Supplementary Materials — The description of the supplementary materials is as follows: This is the detailed information of method validation and method optimization. [file 2184024.f1.docx]

**Sample preparation optimization**

Acetonitrile/ethyl acetate (1: 1, V/V) and acetonitrile/PBS buffer solution (1: 1, V/V) as extracting agent to examine the efficiency. When the acetonitrile/PBS buffer solution was used as the extracting agent, the extraction recoveries for the flumequine enantiomers range from 13.4~17.7%. However, the extraction efficiency of acetonitrile/ethyl acetate were not good. When using ACN/EDTA-Mcllvaine solution as extraction solvent, the extraction efficiency is satisfied. And then, optimize the ratio of ACN and EDTA-Mcllvaine solution, select the ratio of ACN and EDTA-Mcllvaine solution were 10:0, 9:1, 8:2, 7:3, 6:4, 5:5. It was found that when the ratio was 6:4 the recoveries for the two drugs were the highest (Table S1).

Table S1. The average recoveries and RSD% of flumequine at different extraction solvent ratios. (n=3)

|  | Flumequine | | |
| --- | --- | --- | --- |
| Extraction Solvent | Recovery (%) | RSD (%) | CV % |
| ACN/EDTA-Mcllvaine  (10:0, V/V) | 39.70 | 1.73 | 4.37 |
| ACN/EDTA-Mcllvaine  (9:1, V/V) | 37.77 | 5.23 | 13.85 |
| ACN/EDTA-Mcllvaine  (8:2, V/V) | 47.27 | 6.35 | 13.44 |
| ACN/EDTA-Mcllvaine  (7:3, V/V) | 60.04 | 2.25 | 3.75 |
| ACN/EDTA-Mcllvaine  (6:4, V/V) | 77.93 | 1.85 | 2.37 |
| ACN/EDTA-Mcllvaine  (5:5, V/V) | 61.30 | 0.40 | 0.65 |

**Solid phase extraction cartridges optimization.**

The cartridges recoveries experiments were carried out using three solid phase extraction cartridges, Sep-Pak C18 (500 mg, 6 mL), Poly-sery HLB (60 mg, 3 mL), Cleanert PEP (150 mg, 6 mL), respectively. Through the further enrichment of the C18 cartridges filtrate, it was found that there was a large amount of the antibiotic residue in the filtrate, which leads to loss some analytes and low recovery rates, therefore C18 cartridges was unable to absorb flumequine in the extraction solvent. The recoveries of flumequine enantiomers through an HLB cartridges range from 17.8~22.2%. Here, PEP cartridge was chosen as the absorbent for the flumequine enantiomers infusion samples because of its satisfied recovery range from 77~81.8%.

**Recoveries and precision**

The recoveries of method were evaluated by spiking the blank samples at three different concentration levels of the flumequine (10, 50, 100 μg/L for sediment samples and 5, 10, 20 μg/L for water samples). Intra - day precision were obtained by measuring 6 replicates of different matrices at 3 spiked levels within one day. Inter - day precision were obtained for 5 consecutive days. The results of the average recovery, standard deviation and relative standard deviation of the studied flumequine are summarized in Table S2.

Table S2. Spiked average recoveries and relative standard deviations (RSDs) of flumequine. (n=6)

| Sample | Spiked (µg/L) | Flumequine | | | |
| --- | --- | --- | --- | --- | --- |
|  |  | Intra - day | | Inter - day | |
|  |  | Recovery  (%) | RSD | Recovery (%) | RSD |
| Sediment | 10 | 73.6 | 6.3 | 71.7 | 12.5 |
|  | 50 | 74.3 | 12.2 | 73.2 | 5.1 |
|  | 100 | 77.5 | 11.9 | 76.0 | 16.4 |

**Matrix effect**

The matrix effects of each enantiomer of flumequine in sediment samples (Table S3). It was shown that there were matrix-induced effect for the enantiomers of flumequine. The difference in matrix effects of two enantiomers of flumequine are not significant. When all these problems are considered together, we adopted the method of preparing matrix matched standard solution which can eliminate the effect of the matrix and can meet the requirement of residual detection completely.

Table S3. Evaluation of matrix effects of flumequine and 7-hydroxyflumequine in sediment

|  | Calibration curve  without matrix | R^2^ | Calibration curve  with matrix | R^2^ | Matrix effect (%) |
| --- | --- | --- | --- | --- | --- |
| *S*-(-)-flumequine | Y=318748x-281641 | 0.9944 | Y=152208x- 211323 | 0.9915 | -52.2 |
| *R*-(+)-flumequine | Y=344863x-318334 | 0.9919 | Y=187164x- 177560 | 0.9927 | -45.7 |

**Linearity, LOD and LOQ**

We obtained a very good linearity within the concentration range of 1.0 to 200.0 μg/L (1.0, 2.0, 5.0, 10, 20, 50, 100, 200) for each enantiomer of flumequine prepared in the matrix-matched solvent in sediment samples were satisfactory. The calibration curves and correlation coefficients for flumequine enantiomers in different matrix were showed in Table S4.

Table S4. The linear regression equations, regression coefficients (R^2^) and limits of detection (LODs) for enantiomers of flumequine in different samples by HPLC-Q-TOF/MS determination.

| Enantiomers | Samples | Regression equation | R^2^ | LOD (µg/kg or µg/L) |
| --- | --- | --- | --- | --- |
| *S*-(-)-Flumequine | Sediment | Y= 152208x - 211323 | 0.9915 | 5.0 |
| *R*-(+)-Flumequine | Sediment | Y= 187164x- 177560 | 0.9927 | 5.0 |
